# Dermatopontin inhibits WNT signaling pathway via CXXC finger protein 4 in hepatocellular carcinoma

**DOI:** 10.7150/jca.47157

**Published:** 2020-08-29

**Authors:** Shihai Liu, Jing Qiu, Guifang He, Chao Geng, Weitai He, Changchang Liu, Duo Cai, Huazheng Pan, Qingwu Tian

**Affiliations:** 1 Medical Animal Lab, The Affiliated Hospital of Qingdao University, Qingdao, 266000, China.; 2 Department of Stomatology, Qingdao Municipal Hospital, Qingdao, 266071, China.; 3 Department of Clinical Laboratory, The Affiliated Hospital of Qingdao University, Qingdao, 266000, China.; 4 School of Biological Science and Technology, University of Jinan, Jinan, 250022, China.

**Keywords:** Dermatopontin, Wnt pathway, Hepatocellular carcinoma

## Abstract

Hepatocellular carcinoma (HCC) is a major cause of tumor associated deaths globally. Annually, the prevalence of HCC is increasing and the lack of early prognostic indicators manifests a dismal prognosis for HCC patients. A deep understanding of the molecular events that promote HCC progression are required for the design of new diagnostics and therapeutics. Dermatopontin (DPT) is an extracellular matrix protein that regulates the metastatic phenotypes of many cancers. However, the effects of DPT on HCC cell growth remain undefined. In this study, we demonstrate that the exogenous expression of DPT inhibits HCC cell growth both *in vitro* and *in vivo*. Furthermore, we show that DPT regulates CXXC4, which in turn targets c-Myc, EZH2, SOX2 and β-catenin, through its ability to impact Wnt signaling pathway. These data suggest that DPT regulates CXXC4, c-Myc, EZH2, SOX2 and β-catenin, through Wnt signaling to repress HCC proliferation. This highlights DPT as promising target for future HCC diagnostics and therapeutic targets.

## Introduction

Hepatocellular carcinoma (HCC) is a leading cause of liver cancer globally and the 2^nd^ highest cause of cancer associated death in East Asia 1. Current HCC diagnostics are ineffective during the early disease stages, leading to many patients diagnosed with late stage disease leading to a poor prognosis. Identifying new diagnostic and therapeutic targets to improve HCC treatment are urgently required.

Purified from the bovine dermis, dermatopontin (DPT) is an extracellular matrix (ECM) protein rich in tyrosine residues 2. DPT influences the ECM through its ability to enhance fibronectin and collagen fibrillogenesis and through its interactions with decorin and TGF-β, ultimately leading to increase the biological activity of TGF-β 3, 4. Due to its roles in cell-to-cell adhesion and ECM development, DPT regulates multiple physiological processes. The inhibition of DPT is associated with a range of pathologies including systemic sclerosis, uterine leiomyomas, cutaneous fibrosis, and several cancers 5. DPT also inhibits the metastatic properties of oral squamous cell carcinoma (OSCC), though the mechanism(s) of these effects have not been elucidated 6. However, the biological effects of DPT during HCC development have not been defined.

In the current study, we demonstrate that DPT is downregulated in clinical HCC samples and that the exogenous overexpression of DPT inhibits HCC cell proliferation*.* We further demonstrate that DPT regulates CXXC4, which in turn targets the c-Myc, EZH2 and SOX2 axis through its ability to regulate Wnt/β-catenin signaling. These data highlight the suppression of DPT as an important driver of HCC development, and furthers our understanding of HCC development and future avenues of therapeutic strategies.

## Materials and Methods

### Patient tissue

HCC tissues and paired adjacent non-HCC tissues (n = 60 for both) were obtained for patients in our hospital. Informed consent was provided for tissue collection and all protocols were approved by our ethics committee. Patients received surgery with no adjuvant radiotherapy or chemotherapy. Collected tissues were frozen at -80°C prior to analysis. HCC was confirmed in all isolated patient materials independently by two experienced pathologists. The study methodologies conformed to the standards set by the Declaration of Helsinki.

### Cell lines and transfection

HL7702 cells (non-HCC) and Huh7, Hep3B, HCCLM3 and PLC/PRF/5 cells (liver cancer cell lines) were maintained under standard cell culture conditions 7. Briefly, cells were grown in EMEM or DMEM plus 10% Fetal Bovine Serum (FBS), penicillin/streptomycin, and glutamine (2 mM). For DPT overexpression studies, p3×Flag-DPT was purchased from Geneppl technologies (Nanjing, China). For CXXC4 overexpression studies GV141-CXXC4-3XFlag was purchased from GeneChem (Shanghai, China). Silencing studies were performed with shRNAs targeting DPT (shDPT), CXXC4 (shCXXC4), or scrambled controls (shCON). All shRNAs were purchased from GenePharma (Shanghai, China). Transfections were performed using Lipofectamine 3000 (Invitrogen, Carlsbad, CA) as the manufacturer's recommendations.

### 3-(4,5-dimethylthiazol-2-yl)-2,5-diphenyltetrazolium bromide (MTT) assays

For cell viability assessments, HCC cells (~10,000 per well of a 96 well plate) were transfected for 24, 48 and 72 h and subsequently treated with MTT reagent (Sigma-Aldrich, St. Louis, MO) for 2 h. Formazan produced by the cells was then solubilized in DMSO (Sigma-Aldrich, St. Louis, MO). MTT absorbances were measured at 450 nm on a 96-well microplate reader (Bio-Rad, Hercules, CA). All values were normalized to control cells for viability assessments.

### 5-Ethynyl-2'-deoxyuridine (EdU) proliferation assays

EdU incorporation assays were used to measure HCC cell proliferation. Briefly, HCC cells (1 × 10^5^ cells/well) were grown in dishes and treated for 48 h. Cells were then labeled with 50 μM EdU (Beyotime Institute of Biotechnology, Shanghai, China) for 2 h at 37°C. Cells were subsequently fixed in 4% PFA for 30 min, permeabilized with 0.5% Triton X-100 for 30 min and incubated with Apollo reaction cocktail (500 μL/well) for 30 min. Cell nuclei were Hoechst 33342 (Beyotime Institute of Biotechnology, Shanghai, China) stained and cells were imaged via fluorescence microscopy (10 fields of view per treatment). EdU-positive cells were counted on a Nikon, 80i microscope (Nikon, Japan). Average EdU intensities per cell were measured.

### Preparation of cell extracts and Western blotting

Cells were harvested in RIPA buffer plus protease/phosphatase inhibitors and protein content were quantified via Bio-Rad assays. In total, 20-50 μg of each sample were resolved by SDS-PAGE and proteins were semi-dry transferred onto PVDF membranes (Millipore Corp., Bedford, MA) and blocked for 1 h in Blotto A (5% milk in TBS-T), followed by labeling for 1 h with Blotto A containing anti-DPT (Cat. ab255823, Abcam, Cambridge, MA), anti-EZH2 (Cat. ab191250, Abcam, Cambridge, MA), anti-β-catenin (Cat. #8480, CST, Danvers, MA), anti-SOX2 (Cat. #4900, CST, Danvers, MA), c-Myc (Cat. ab39688, Abcam, Cambridge, MA), anti-p-GSK3β (Cat. #5558, CST, Danvers, MA), CXXC4 (Cat. ab105400, Abcam, Cambridge, MA) and anti-GAPDH (Cat. ab8245, Abcam, Cambridge, MA) antibodies. Blots were washed and labeled in Blotto A containing the appropriate HRP-conjugated secondary antibodies (CST, Danvers, MA). Membranes were washed in TBS-T. The ECL system was used for band visualization and values were quantified and normalized to GAPDH as a housekeeper control.

### Luciferase reporter assays

The Promega dual luciferase system was used for TOPFlash/FOPFlash reporter assays (Promega, Madison, WI). Briefly, cells were co-transfected with TOPFlash/FOPFlash for 24 h and lysed in commercially available Dual-Luciferase Reporter Assay buffer. Firefly and Renilla activity were then assessed on a luminometer. Data presented is firefly luciferase activity after normalization with Renilla luciferase.

### Quantitative real time Polymerase Chain Reaction (qPCR) analysis

RNA was purified using commercial RNeasy Mini kits (Qiagen, Hilden, Germany) according to the manufacturer's recommendations. CDNA synthesis kits (iScript, Bio-Rad, Hercules, CA) were used for first stand synthesis. Gene expression profiles of Wnt related genes (n = 84) were assessed using the Wnt Signaling Pathway RT^2^ Profiler PCR Arrays (Cat. PAHS-043ZR, Qiagen) on a 7500 Sequence Detection System (Applied Biosystems, Foster City, CA) plus recommended controls. Relative gene expression was assessed using a Qiagen RT^2^ Profiler PCR Array via the CT method. Values were normalized to GAPDH as a reference gene. PCR arrays, whose threshold cycle above the 35th cycle, were excluded. Data were analyzed using Qiagen web-based data analysis.

### Clonogenic assays by soft agar

To assess the colony formation capacity of HCC cells, cells were harvested in trypsin and seeded (2 × 10^3^ cells per 6-well plate) in complete DMEM containing 0.3% and 0.6% agar. Cells were then maintained under standard culture conditions for a 15 days period, at which time, colonies were fixed in 70% ethanol and imaged. Colonies ≥ 50 cells were deemed positive.

### Cell imaging by immunofluorescence (IF)

For IF analysis, cells were fixed in 4% PFA, treated with pepsin, and dehydrated using a gradient ethanol series. Cells were then permeabilized in Triton X-100, blocked in PBS plus goat serum, and probed with anti-β-catenin antibodies (Cat. #8480, CST, Danvers, MA) overnight. Cells were washed and labeled with the appropriate fluorescently conjugated secondary antibodies. Nuclei were counter-stained with 4',6-diamidino-2-phenylindole (DAPI) and imaged on a Leica TCS SPE confocal and DM 5500 Q microscope (Leica Microsystems, Germany). For all data, representative confocal images were shown.

### Co-Immunoprecipitation (Co-IP)

Cells (2 × 10^6^) were lysed in Co-IP buffer containing Tris-HCl (pH 7.5), 150 mM NaCl, 1% Nonidet P-40, and 10% glycerol plus protease/phosphatase inhibitors. Lysates were pre-cleared and labeled with anti-flag antibodies (Cat. #14793, CST, Danvers, MA) or anti-Dvl-1 (Cat. sc-8025, Santa Cruz, CA) overnight at 4°C with mixing. Protein A/G conjugated to agarose (Santa Cruz, CA) was then added for 2 h and pelleted through centrifugation. Control samples contained rabbit IgG alone. Beads were washed in IP buffer (×3 times) and mixed with 2 × loading buffer. Samples were boiled for 5 min and assessed by Western blotting analysis 8. Representative Co-IPs are shown throughout.

### Tumor assessments

Tumor growth assays were performed in athymic BalB/C nude mice (obtained from the Beijing Vital River Laboratory). HCCLM3 cells (1 × 10^6^) were injected into the left dorsal flank (n = 6 per treatment) and monitored daily. Upon tumor sizes of 40 to 50 mm^3^ being observed, mice were treated for 5 days. An electronic digital caliper was used to assess tumor growth characteristics and tumor volumes were calculated using the equation: tumor size=*a*×*b^2^*/2*, a:* larger and *b:* smaller of the dimensions. Mice were sacrificed 24 d post-injection and tumor characteristics were examined.

### Statistics

All statistical analyses were carried out using SPSS version 18.0 (IBM Corp., Armonk, NY). The associations between DPT expression and the clinicopathological characteristics of the patients were analyzed using the Chi-squared test. Bivariate correlations between study variables were calculated using the Spearman's rank correlation coefficient. Survival curves were plotted using the Kaplan-Meier method and compared using the log-rank test. Data are shown as the mean ± SD. *P <* 0.05 indicated a significant difference between treatment groups.

## Results

### DPT is downregulated in HCC tissues and correlates with patient survival

**Figure 1:**
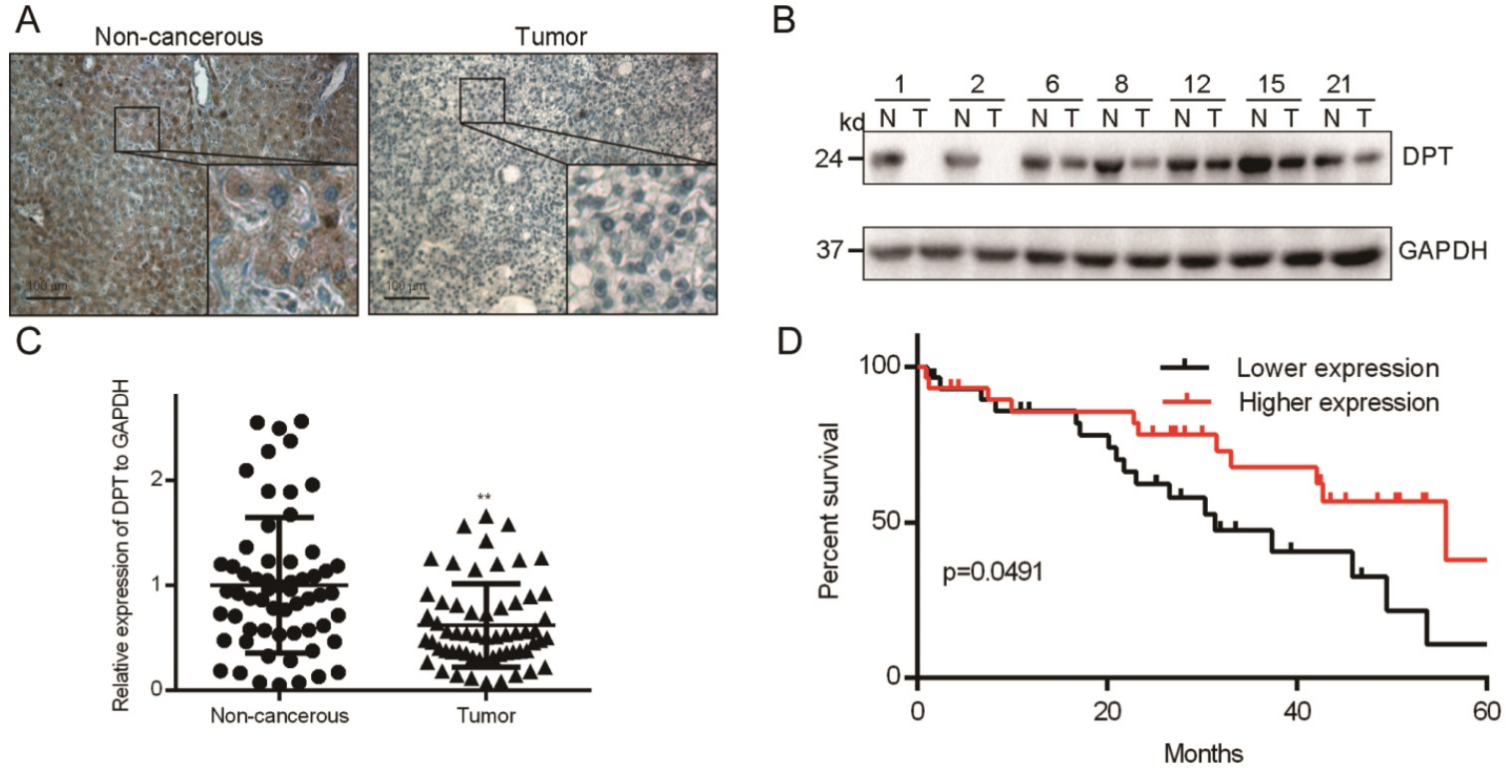
** DPT levels in HCC tissue. (A)** IHC analysis.** (B)** Representative Western blotting and** (C)** qPCR analysis of DPT levels in HCC vs. non-cancerous tissue.** (D)** Analysis of overall survival of patients with different DPT expression. All data are shown relative to GAPDH expression and were compared using a Student's *t* test.

A deep understanding of the molecular events that promote HCC progression are required for the effective development of new therapeutics. DPT is an extracellular matrix protein that regulates the metastatic phenotypes an array of human neoplasms. We initially assessed the expression of DPT in Cancer transcription analysis on TCGA samples from UALCAN database (http://ualcan.path.uab.edu/index.html) and GEPIA database (http://gepia.cancer-pku.cn/). UALCAN showed that DPT was downregulated expressed in HCC compared with that in normal liver tissues (Figure S1A). We got the similar results in GEPIA database (Figure S1B). DPT expression was positively correlated with the clinical stage and pathological grade (Figure S1C and S1D). Then, we assessed DPT expression in 60 paired HCC tissues obtained from patients in our hospital who underwent surgery. HCC was confirmed in all samples by an experienced panel of pathologists. From IHC analysis, DPT had a cytoplasmic distribution (Figure 1A) indicative of a soluble protein. Western blotting analysis further showed that the total levels of DPT expression in surgical tissues from non-cancerous (N) were higher than that of HCC tumor tissue (T) (Figure 1B). To investigate the frequency of DPT regulation in HCC, we examined the expression using qPCR in 60 fresh-frozen HCC tissues, including 52 cases at clinical stage I and II, 8 cases at clinical stage III and IV (Table 1). We observed significant downregulation of DPT in HCC tissues (Figure 1C). Then we analyzed the association between DPT and the clinicopathological features of HCC. As shown in Table 2, strong associations were observed between DPT expression and age (*P =* 0.048) and alcoholism (*P =* 0.029). However, the expression of DPT was not associated with gender (*P =* 0.648), liver cirrhosis (*P =* 0.570), AFP level (*P =* 0.855), ALT (*P =* 0.128), AST (*P =* 0.144), tumor number (*P =* 0.206), tumor size (*P =* 0.993), portal vein invasion (*P =* 0.282) or TNM stage (*P =* 0.156). Spearman analysis of correlation between DPT and clinicopathological features revealed that the expression of DPT was significantly correlated with age (*P =* 0.049) and alcoholism (*P =* 0.029) (Table 3). Kaplan-Meier survival curves demonstrated that the overall survival of patients with high expression of DPT was significantly longer than those with low DPT expression (Figure 1D, *P =* 0.0491). Collectively, these results indicate that downregulation of DPT in primary HCC patients correlate with poor survival.

### DPT overexpression inhibits HCC cell growth *in vitro* and tumorigenicity *in vivo*

**Figure 2:**
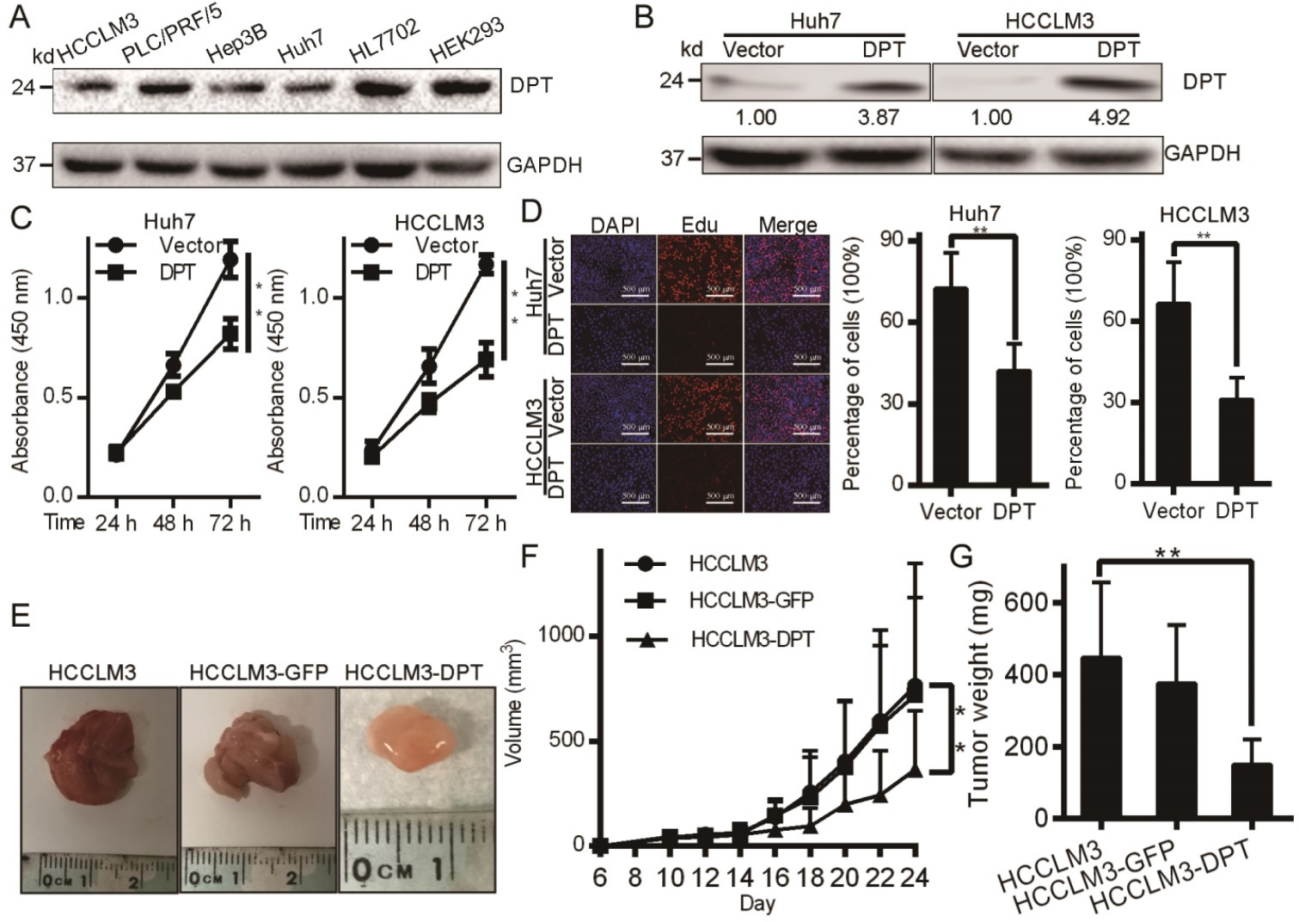
** DPT inhibits HCC cell growth and tumorigenicity. (A)** DPT levels in the indicated HCC cell lines.** (B)** DPT overexpression assessed by Western blotting.** (C)** Cell growth and MTT assays in response to exogenous DPT overexpression.** (D)** HCC cell lines were treated with DPT for 48 h, EdU treated and fixed. The incorporation of EdU was assayed using a BeyoClick Kit. Representative images are shown. **(E-G)** Tumor growth curves and weights in response to exogenous DPT expression. Data were analyzed using a Student's t test. Data are the mean ± SD. **P < 0.01.

We next assessed the levels of DPT in four independent HCC cell lines by Western blotting analysis. Figure 2A shows that DPT expression was downregulated in all HCC cells assessed. DPT overexpression experiments were next performed in Huh7 and HCCLM3 cells through the exogenous expression of DPT overexpression vectors (Figure 2B). Following DPT overexpression, cell growth and viability significantly declined in Huh7 and HCCLM3 cells vs. control groups (Figure 2C-D, *P <* 0.05). We next injected 1 × 10^6^ cells into nude mice and monitored mouse weights and tumor growth (Figure 2E). We found that HCCLM3-DPT xenografts were of reduced weight and size compared to control groups (Figure 2F-G, *P <* 0.05). These data indicated that DPT overexpression inhibits HCC cell growth *in vitro* and HCC tumor formation *in vivo.* To sum up, this highlighted DPT as a potential therapeutic target in HCC.

### DPT overexpression in HCC cells displays decreased characteristics of cancer stem cells (CSCs)

**Figure 3:**
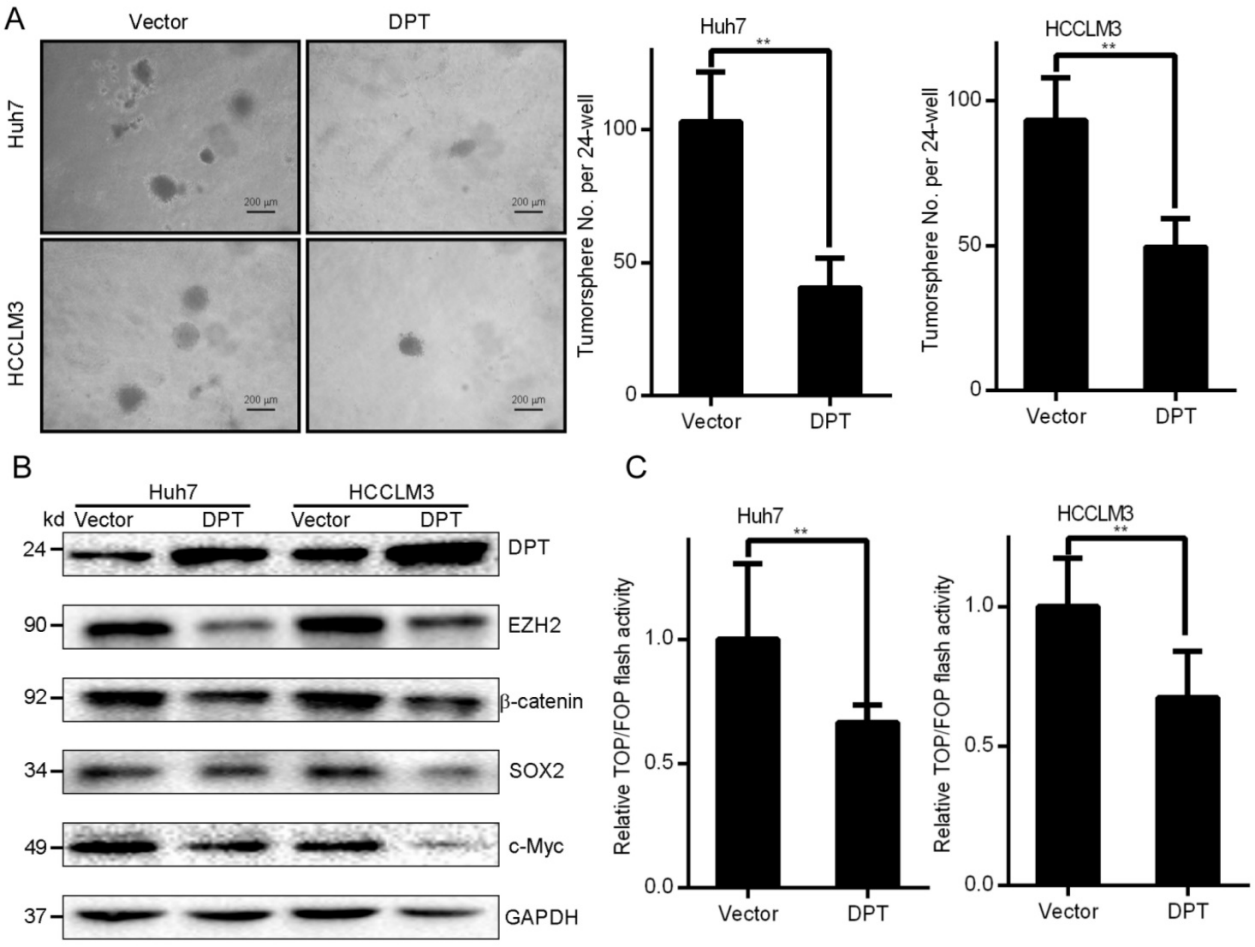
** Exogenous DPT expression inhibits the CSC phenotypes of HCC. (A)** Representative images of the tumor spheres in response to DPT overexpression. **(B)** Western blotting analysis.** (C)** Huh7 and HCCLM3 cells overexpressing DPT were transfected with TOP/FOPFlash reporter plasmids. Luciferase activity was assessed after 24 h of transfection. Data are the mean ± SD of triplicate experiments. Data were compared through a Student's t test. **P < 0.01.

Small populations of CSCs drive HCC tumor growth. CSCs undergo self-renewal *in vitro*. Cells exogenously expressing DPT or control cells were cultured in serum-free medium to permit the growth of tumor spheres (Figure 3A). In DPT overexpressing cells, colony formation in soft agar was found to be inhibited vs. control groups (Huh7 and HCCLM3). These data indicated that DPT inhibited the self-renewal capacity of HCC cells. Stem cells-related transcription factors are key to self-renewal capacity. In DPT overexpressing cells, the levels of SOX-2, EZH2, and c-Myc were significantly suppressed (Figure 3B) in addition to a loss of Wnt/β-catenin signaling components evidenced through the levels of TOP-Flash reporter activity in Huh7 and HCCLM3 cells vs. control groups (Figure 3C, *P <* 0.15) 9. The culmination of these data highlight that the overexpression of DPT in HCC cells leads to a loss of CSC characteristics.

### DPT overexpression inhibits Wnt/β-catenin signaling

**Figure 4:**
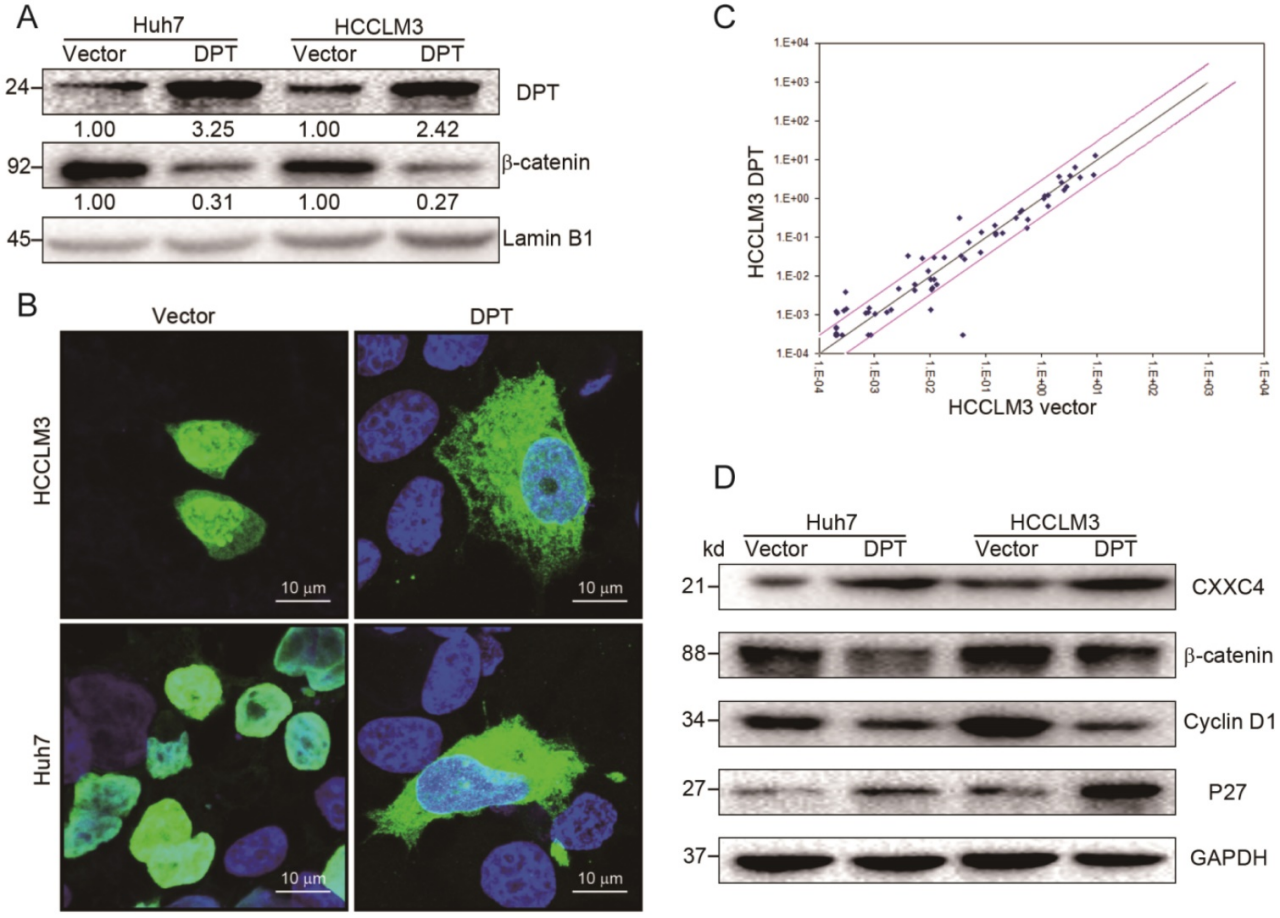
** Overexpression of DPT inhibits Wnt/β-catenin signaling. (A)** β-catenin expression in the nucleus in response to DPT-overexpression.** (B)** Immunofluorescence analysis of β-catenin levels in DPT-overexpressing HCC cells. Cells were fixed in 4% PFA, treated with pepsin, and dehydrated using a gradient ethanol series. Cells were permeabilized in Triton X-100, blocked in PBS plus goat serum, and probed with anti-β catenin antibodies and the appropriate secondary antibodies. Cell nuclei were counter-stained with DAPI. Representative confocal images were shown.** (C)** Expression of Wnt-related genes. Black line: fold-change in gene expression of 1. Pink line: desired fold change in gene expression threshold. **(D)** Expression of CXXC4, β-catenin, Cyclin D1 and P27 assessed by Western blotting. Data are the mean ± SD and were compared using a student's t-test. *P < 0.05.

The effects of DPT overexpression were next assessed by Western blotting and immunofluorescent analysis. We observed a loss of β-catenin expression and nuclear accumulation (Figure 4A-B) in cells overexpressing DPT. To provide mechanistic insight into these effects, we analyzed the gene expression profiles of HCCLM3 cells overexpressing DPT (HCCLM3-DPT) vs. mock cells (HCCLM3-vector) with a focus on Wnt-related genes, assessed through commercially available qPCR arrays assay.

We found that DPT treatment led to the upregulation of 9 key Wnt-related genes, including WNT5A, WNT16, CXXC4, WNT11, CCND2, TCF7, WNT2B, RHOU and PPARD (≥ 2-fold increase, Figure 4C, Table S1). The expression of EP300, BCL9, WNT8A, PORCN, PYGO1, WNT10A, MYC, WNT7A and CSNK1A1 decreased in response to DPT (Figure 4C, Table S1). Of the upregulated genes, CXXC4 (CXXC finger protein 4) was validated by Western blotting (Figure 4D). The expression of p27 also increased in response to DPT, whilst the expression of Cyclin D1 and β-catenin declined. These data suggest that DPT acts as a tumor suppressor in HCC through the inactivation of pro-oncogenic Wnt/β-catenin signaling pathway.

### CXXC4 functions as a tumor suppressor gene in HCC

**Figure 5:**
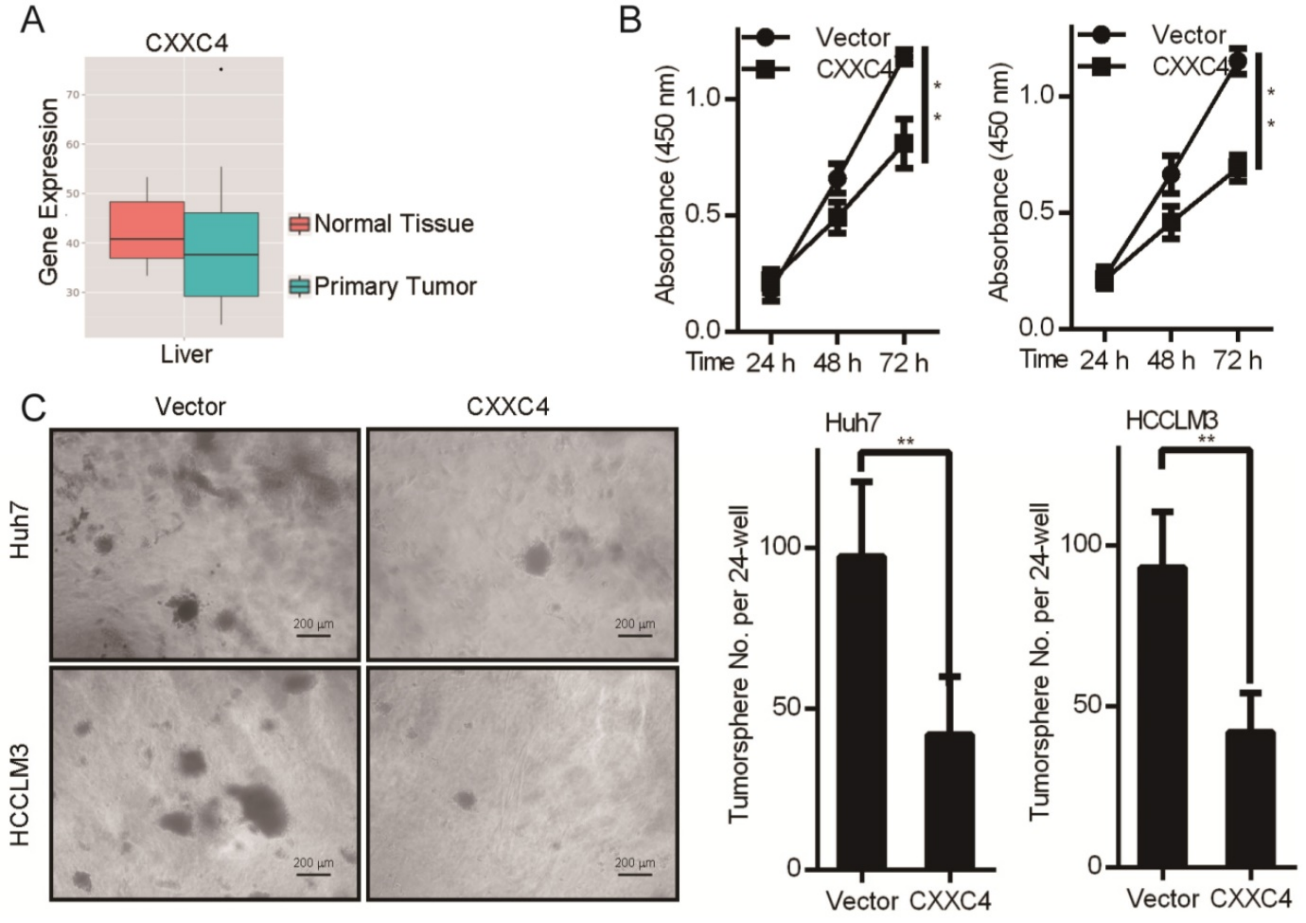
** CXXC4 inhibits HCC cell proliferation and colony formation. (A)** Metabolic gEne RApid Visualizer (MERAV) boxplots for CXXC4 expression in normal vs. HCC tissue.** (B)** MTT assays in cells overexpressing CXXC4. Values: mean ± SD of absorbance at 450 nm (n = 5).** (C)** Colony formation assays. Data are the mean ± SD (n = 3); *P < 0.05, **P < 0.01.

We next explored the tumor functions of CXXC4 in HCC. Boxplots illustrating the expression of CXXC4 in normal vs. HCC tissue were produced through MERAV analysis (Figure 5A). The median expression of CXXC4 was lower in healthy vs. HCC tissues. CXXC4 overexpression also led to a loss of Huh7 and HCCLM3 cell growth as assessed by MTT viability assays (Figure 5B). To further examine the effects of CXXC4 on HCC cells, both cell-types were engineered to overexpress CXXC4 through soft agar assays (Figure 5C). In response to CXXC4 overexpression, the growth of all assessed HCC cell lines was inhibited (Figure 5C). These data demonstrated that CXXC4 was an important tumor suppressor in HCC.

### CXXC4 inhibits Wnt signaling in HCC

**Figure 6:**
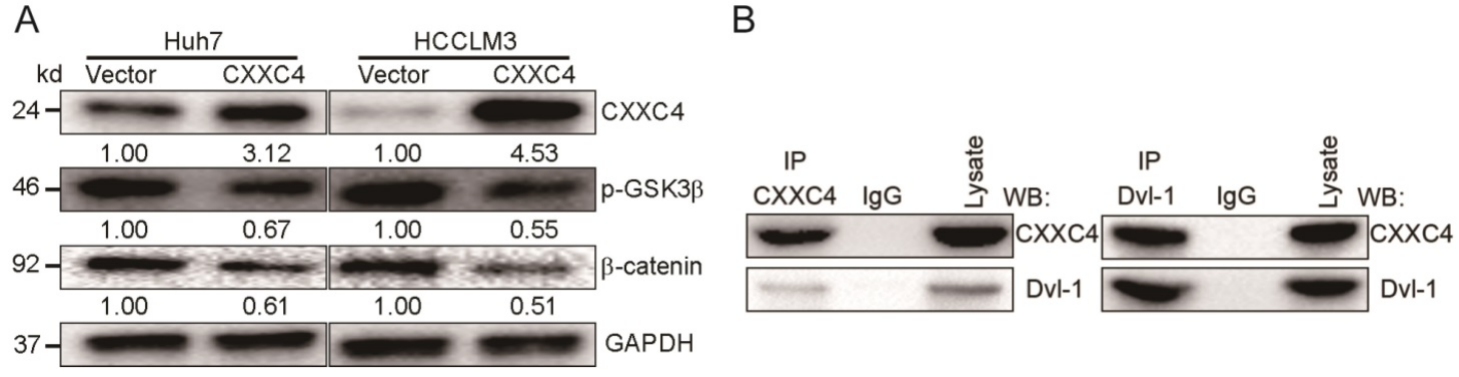
** CXXC4 inhibits Wnt signaling in HCC cells. (A)** Levels of β-catenin and p-GSK-3β in Huh7 cells in response to CXXC4 overexpression assessed by western blotting.** (B)** Cells (2 × 10^6^) were lysed in Co-IP buffer and labeled with anti-flag or anti-Dvl-1 antibodies. Protein A/G conjugated to agarose was then added for 2 h and pelleted through centrifugation. Control samples contained rabbit IgG alone. Beads were washed in IP buffer and mixed with 2 × loading buffer. Samples were boiled for 5 min and assessed by Western blotting analysis. Representative IPs showing the Dvl-1 interaction with CXXC4 are shown.

CXXC4 is a negative regulator of Wnt/β-catenin signaling in renal cell carcinoma (RCC) and gastric cancer in the previously report 10. We assessed the contribution of CXXC4 to Wnt signaling in HCC. Exogenous CXXC4 expression reduced β-catenin expression (Figure 6A) and p-GSK-3β levels (Figure 6A). In this study, we observed a CXXC4 and Dvl-1 interaction through Co-IP assays in HCCLM3 cells (Figure 6B) confirming this mechanism in HCC. This furthers our understanding of the molecular mechanisms governing HCC progression.

### DPT inactivates Wnt signaling through upregulating CXXC4

**Figure 7:**
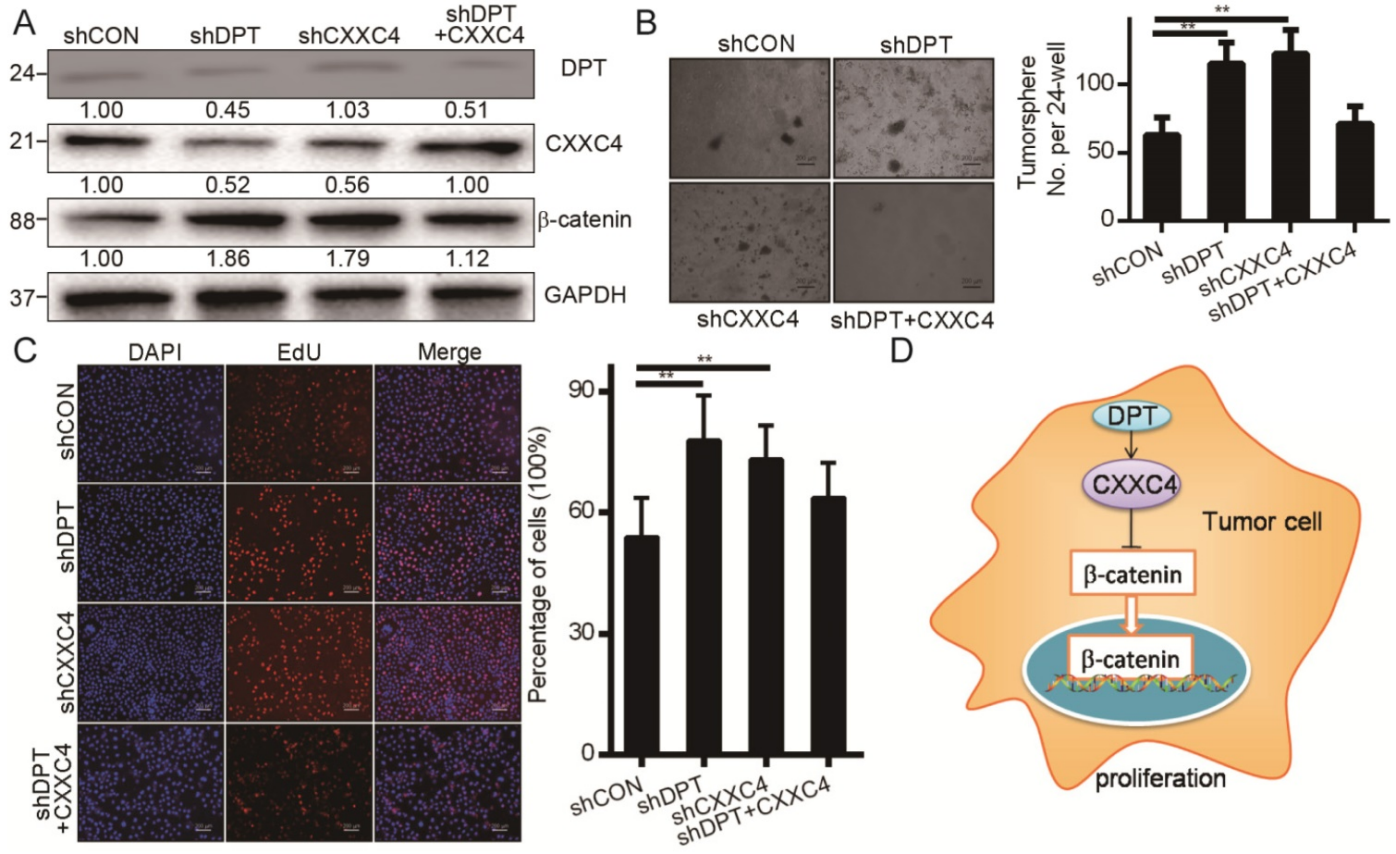
** CXXC4 inactivates Wnt through CXXC4. (A)** CXXC4 and β-catenin levels in shDPT, shCXXC4 or shDPT cells assessed through Western blotting analysis. **(B)** Effect of shDPT, shCXXC4 or shDPT and CXXC4 on colony formation.** (C)** Huh7 cells were treated with shDPT, shCXXC4 or shDPT and CXXC4 for 24 h and stained with EdU and DAPI.** (D)** Proposed model: Indicating that DPT upregulates the expression of CXXC4. CXXC4 could stabilize the degradation of β-catenin and reduce β-catenin expression.

We next assessed whether DPT inhibits Wnt signaling and HCC progression through its effects on CXXC4. We found that the downregulation of DPT enhanced β-catenin expression in HCCLM3 cells (Figure 7A), whilst the silencing of CXXC4 induced β-catenin levels (Figure 7A). We also found that the inhibitory effects of DPT shRNA could be reversed by the overexpression of CXXC4 (Figure 7B), suggesting that DPT inactivates Wnt signaling to promote HCC carcinogenesis. Cell growth and Wnt were inhibited by the exogenous expression of CXXC4 (Figure 7B-C). CXXC4 shRNA cells were able to maintain their inhibition of cell growth and β-catenin upon simultaneous CXXC4 activation (Figure 7A-7C). The culmination of these data confirmed that DPT inhibits Wnt signaling in HCC through its ability to upregulation of CXXC4 expression (Figure 7D).

## Discussion

HCC is now the 2^nd^ leading cause of cancer associated death globally and a major cause of mortality in China and across the world 11. Whilst anti-HCC therapies have improved and evolved, the 5-year overall survival rates in HCC patients remain dismal, due to the high levels of recurrence and metastasis following surgery 12, 13. Therefore, an improved understanding of HCC progression and metastasis is therefore required to improve HCC therapy.

It has been reported that DPT competes with decorin for its interaction with TGF-β 3, which regulates a range of pathological processes, including tumor progression, growth and survival 14. We found that the overexpression of DPT suppresses HCC cell proliferation. However, the effects of DPT on HCC cell growth and its relationship to Wnt signaling is undefined. Emerging evidence suggests the existence of a cross-talk mechanism between TGF-β, integrins and the ECM 15. Further assessments of the contribution of DPT to this signaling axis will enhance our understanding of the mechanisms of HCC progression.

Canonical Wnt signaling regulates HCC cell self-renewal and drug-resistance, widely contributing to the poor prognosis of HCC 16, 17. Despite the incomplete statistics, approximately 30% of HCC cases display aberrant Wnt activation 18. The dysregulation of the Wnt/β-catenin axis is a known oncogenic driver, and represents an attractive therapeutic strategy for HCC 19. DPT regulates the progression and development of several human cancers 2, 6, 20. DPT is downregulated during cancer malignancy, as shown by Yamatoji and colleagues in human OSCC. Guo and coworkers additionally reported that DPT expression is downregulated in papillary thyroid cancer 21. In this study, using Western blotting analysis, bioinformatics, qPCR and IHC assessments, DPT expression was shown for the first time to be suppressed in HCC.

Aberrant Wnt/β-catenin signaling due to genetic changes in APC or CTNNB1 is a driver of an array of human malignancies 22, 23. However, aberrant cellular CTNNB1 translocation occurs in up to 90% of HCC cases but CTNNB1 mutations only occur in 2.8%-44% of cases, suggesting other mechanisms of Wnt deregulation in HCC 24. The tumor suppressor functions of CXXC4 are also well-characterized and CXXC4 inhibits Wnt/β-catenin signaling through its interaction with Disheveled 25-27. Aberrant Wnt signaling pathway activation predicts poor cancer patient prognosis 28. EZH2 performs a pivotal and essential function in maintaining cancer properties in Wnt/β-catenin signaling activation 29. SOX2 has a crucial role in the proliferation of cancer Cells through c-MYC in Wnt/β-catenin pathway 30. In this study, we identified DPT as a mechanism to inactivate Wnt signaling through CXXC4, representing a novel epigenetic mechanism of HCC mediated Wnt signaling (Figure 7D).

In summary, our data provide the first evidence of the epigenetic upregulation of DPT in HCC and its effects on Wnt signaling. We show that the DPT mediated repression of CXXC4 leads to the inhibition of constitutively active Wnt/β-catenin, consistent with studies highlighting the ability of EZH2 to promote carcinogenesis through the repression of ADRB2, CDH1, PSP94 and DAB2IP and other tumor suppressors 25. These data highlight how epigenetic regulation in addition to genetic changes regulates the aberrant activation of Wnt/β-catenin signaling in HCC.

In summary, we have revealed CXXC4 as a target for DPT and its suppression during HCC development. We show that DPT functions as a tumor suppressor in HCC and inhibits Wnt signaling through CXXC4 mediated β-catenin degradation.

## Conclusions

Here, we reveal that DPT is poorly expressed in HCC tissues and cells, and that its recovery leads to anti-HCC effects. Mechanistically, we reveal that DPT suppresses Wnt signaling through its activation of CXXC4, regulating downstream targets including EZH2, β-catenin, SOX2 and c-Myc. These findings highlight the anti-HCC effects of DPT revealing its potential as a novel therapeutic target for HCC.

## Supplementary Material

Supplementary figures and tables.

## Figures and Tables

**Table 1:** Clinicopathological characteristics of patient samples and expression of DPT in HCC

Characteristics	No. of case (%)
**Age**	
<50	14 (23.3)
≥50	46 (76.7)
**Gender**	
Male	49 (81.7)
Female	11 (18.3)
**Alcoholism**	
Yes	16 (26.7)
No	44 (73.3)
**Liver cirrhosis**	
Yes	35 (58.3)
No	25 (41.7)
**AFP (ng/L)**	
<200	40 (66.7)
≥200	20 (33.3)
**ALT (U/L)**	
<60	42 (70.0)
≥60	30 (30.0)
**AST (U/L)**	
<40	40 (66.7)
≥40	20 (33.3)
**Tumor number**	
Single	36 (60.0)
Multiple	24 (40.0)
**Tumor size**	
<5cm	31 (51.7)
≥5cm	29 (48.3)
**Portal vein invasion**	
Yes	13 (21.7)
No	47 (78.3)
**TNM stage**	
I+II stage	52 (86.7)
III+IV stage	8 (13.3)

HCC, hepatocellular carcinoma; AFP, α-fetoprotein; ALT, alanine aminotransferase; AST, aspartate aminotransferase; TNM, tumor node metastasis.

**Table 2:** Correlation between DPT expression and clinicopathologic characteristics of HCC patients

Characteristics DPT	DPT expression		
	Low or none, no. cases	High, no. cases	*p* value
**Age**			
<50	4	10	**0.048**
≥50	27	19	
**Gender**			
Male	26	23	0.648
Female	5	6	
**Alcoholism**			
Yes	12	4	**0.029**
No	19	25	
Liver cirrhosis			
Yes	17	18	0.570
No	14	11	
**AFP (ng/L)**			
<200	21	19	0.855
≥200	10	10	
**ALT (U/L)**			
<60	19	23	0.128
≥60	12	6	
**AST (U/L)**			
<40	18	22	0.144
≥40	13	7	
**Tumor number**			
Single	21	15	0.206
Multiple	10	14	
**Tumor size**			
<5cm	16	15	0.993
≥5cm	15	14	
**Portal vein invasion**			
Yes	5	8	0.282
No	26	21	
**TNM stage**			
I+II stage	25	27	0.156
III+IV stage	6	2	

*P* values were calculated using chi-square test. Bold numbers indicate significant differences (*P <* 0.05). HCC, hepatocellular carcinoma; AFP, α-fetoprotein; ALT, alanine aminotransferase; AST, aspartate aminotransferase; TNM, tumor node metastasis.

**Table 3:** Spearman analysis of correlation between DPT and clinicopathological

Variables	DPT expression level	
	Spearman correlation	*p* value
Age (years, <50 vs. ≥50)	-0.255	0.049
Gender (male/female)	0.059	0.655
Alcoholism (yes/no)	-0.282	0.029
Liver cirrhosis (yes/no)	0.073	0.578
AFP (ng/L, <200 vs. ≥200)	0.024	0.858
ALT (U/L, <60 vs. ≥60)	-0.197	0.132
AST (U/L, <40 vs. ≥40)	-0.189	0.149
Tumor number (single/multiple)	0.163	0.212
Tumor size (cm, <5 vs. ≥5)	-0.001	0.993
Portal vein invasion (yes/no)	0.139	0.290
TNM stage (I+II vs III+IV)	-0.183	0.161

Bold numbers indicate significant differences (*P <* 0.05). HCC, hepatocellular carcinoma; AFP, α-fetoprotein; ALT, alanine aminotransferase; AST, aspartate aminotransferase; TNM, tumor node metastasis.
