# Supplementary material for: Dermatopontin inhibits WNT signaling pathway via CXXC finger protein 4 in hepatocellular carcinoma
Source: J Cancer. 2020 Aug 29;11(21):6288–98. doi: 10.7150/jca.47157 (PMC7532498; doi:10.7150/jca.47157)
Supplement: Supplementary file 1 — Supplementary figures and tables. [file jcav11p6288s1.pdf]

Dermatopontin inhibits WNT signaling pathway via CXXC finger protein 4 in hepatocellular carcinoma

Shihai Liu[1], Jing Qiu[2], Guifang He[1], Chao Geng[3], Weitai He[4], Changchang Liu[1], Duo Cai[1], Huazheng Pan[3]*, Qingwu Tian[3]*

1 Medical Animal Lab, The Affiliated Hospital of Qingdao University, Qingdao, 266000, China

2 Department of Stomatology, Qingdao Municipal Hospital, Qingdao, 266071, China

3 Department of Clinical Laboratory, The Affiliated Hospital of Qingdao University, Qingdao, 266000, China

4 School of Biological Science and Technology, University of Jinan, Jinan, 250022, China

*Corresponding to author:
Huazheng Pan, Ph. D.
Department of Clinical Laboratory
The Affiliated Hospital of Qingdao University
No. 16, Jiangsu Road, Qingdao 266000, P.R. China
Tel: +86 532 82911773
Fax: +86 532 82911773
E-mail address: panhuazheng@126.com (H. Pan)

Qingwu Tian, Professor
Department of Clinical Laboratory
The Affiliated Hospital of Qingdao University
No. 1677, Wutaishan Road, Qingdao 266000, P.R. China
Tel: +86 532 82919388
Fax: +86 532 82919388
E-mail address: tianqingwu1970@163.com (Q. Tian)

Supplementary Figure S1: Low expression of DPT is positively correlated with poor prognosis in HCC patients. (A) Expression level of DPT in primary tumors and normal liver tissues based on TCGA dataset through UALCAN. $P<0.05$; (B) Expression level of DPT in primary tumors and normal liver tissues based on TCGA dataset through GEPIA. $P<0.05$; (C) Analysis on the expression levels of DPT in TCGA HCC samples based on individual clinical stage. (d) Analysis on the expression levels of DPT in TCGA HCC samples based on individual pathological grade.

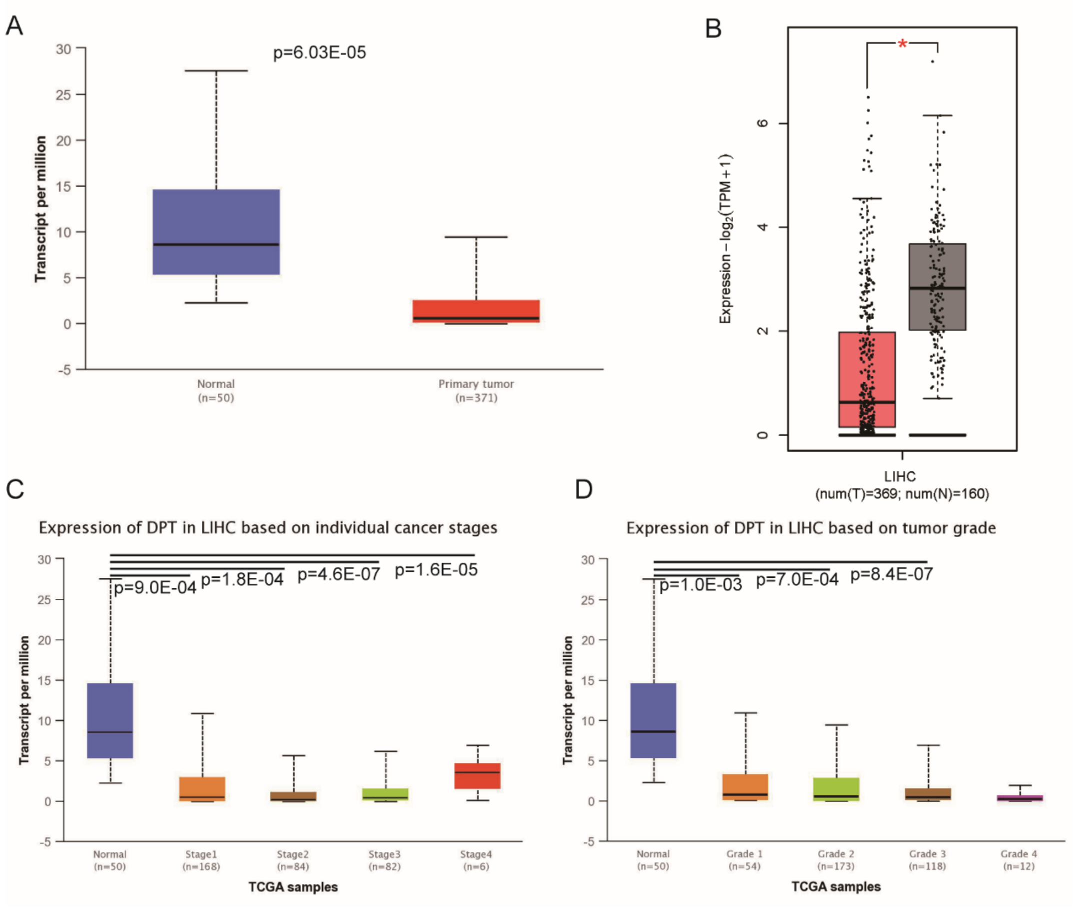

Supplementary Table S1: Expression of RNAs in DPT overexpression HCCLM3 vs HCCLM3 vector control as determined by real time PCR arrays.

Gene name	Fold change (DPT treatment vs control)
WNT5A	12.94
WNT16	8.19
CXXC4	5.87
WNT11	5.18
CCND2	4.74
TCF7	4.54
WNT2B	2.57
RHOU	2.34
PPARD	2.23
EP300	-2.06
BCL9	-2.08
WNT8A	-2.15
PORCN	-2.36
PYGO1	-2.49
WNT10A	-2.74
MYC	-3.11
WNT7A	-7.44
CSNK1A1	-124.16
